# A case of dermatofibrosarcoma protuberans with neurofibromatous change

**DOI:** 10.1093/jscr/rjab472

**Published:** 2021-12-11

**Authors:** Kazuhiro Kudoh, Chieko Itabashi, Eiichi Arai, Shusa Ohshika, Hiroki Mizukami

**Affiliations:** Department of Pathology and Molecular Medicine, Hirosaki University, Hirosaki, Japan; Department of Pathology and Molecular Medicine, Hirosaki University, Hirosaki, Japan; Department of Pathology, Saitama Medical University International Medical Center, Saitama, Japan; Department of Orthopedic Surgery, Hirosaki University, Hirosaki, Japan; Department of Pathology and Molecular Medicine, Hirosaki University, Hirosaki, Japan

## Abstract

A 31-year-old man with posterior neck mass visited a hospital. The mass recurred four times on the same location during the past 6 years. Needle biopsy diagnosis was suspicious for benign stromal tumor. Tumor excision was performed 3 months after the biopsy. The tumor size was 8.3 × 4.5 cm and was located at subcutaneous tissue. Histologically, main tumor cells showed comma-shaped nuclei, which are same as neurofibroma. Immunohistochemically, tumor cells were positive for vimentin, CD34, but were negative for S-100. Fluorescence *in situ* hybridization analysis disclosed a split signal of PDGFB gene. Reverse transcriptase-polymerase chain reaction clarified COL1A1 exon 47/PDGFB exon 2 chimeric gene. Final diagnosis was dermatofibrosarcoma protuberans (DFSP) with neurofibromatous change. DFSP with neurofibromatous change is rare and could be misdiagnosed as benign tumor, especially in a biopsy specimen. Molecular diagnosis is a promising aid in a challenging case and in biopsy specimens.

## INTRODUCTION

Dermatofibrosarcoma protuberans (DFSP) is an intermediate grade malignant fibroblastic neoplasm [1]. Whereas neurofibroma-like change occurs infrequently in this tumor, hitherto, few literatures mention the significance of this finding on pathological diagnosis [2]. This histological variation can lead to misdiagnosis as benign lesions, especially in a biopsy specimen. Herein, we report a case of DFSP with neurofibromatous change, which was initially diagnosed as benign tumor.

## CASE REPORT

**Figure 1:**
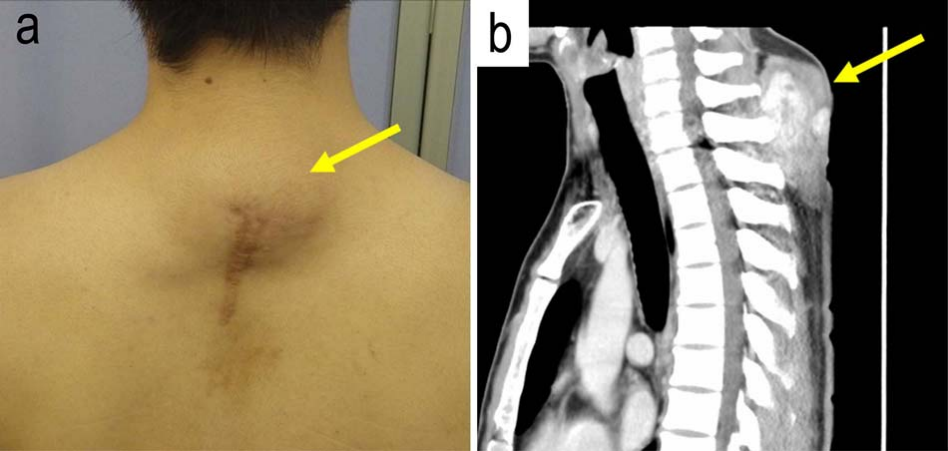
The gross and magnetic resonance imaging (MRI) finding of the tumor; a subcutaneous tumor beneath operation scar was located on the posterior neck of the patient (**a**); MRI study visualized a mass in the subcutis of the neck (**b**).

A 31-year-old man was aware of posterior neck mass. The patient had a past history of tumor that had emerged on the same location three times during the past 6 years. Although tumor excisions were performed in each time, pathological examination had not been done. The fourth emerged mass was located beneath the operation scar on the posterior neck. The maximum diameter of the mass was 6.0 cm (Fig. 1a). Magnetic resonance imaging visualized a solid mass with ambiguous border in the subcutis of the neck (Fig. 1b).

**Figure 2:**
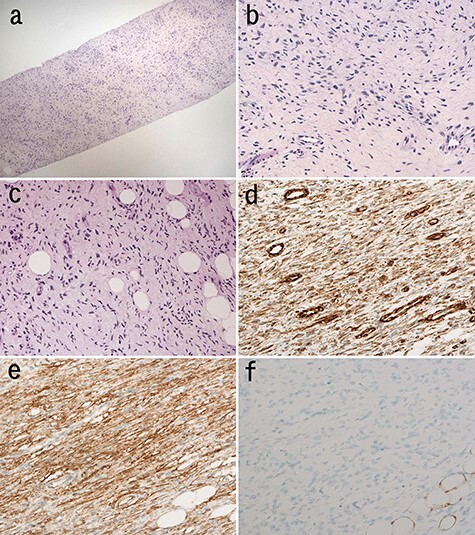
Microscopic findings of the needle biopsy; myxomatous stroma intervened at low magnification of the biopsy; (H&E staining) (**a**); spindle cells proliferated haphazardly (**b**); cellular atypia of these cells is mild; (H&E staining) (b); tumor cells infiltrated into adipose tissue in the lesion (**c**); tumor cells with wavy nuclei were also seen (H&E staining) (c); immunohistochemically, tumor cells were positive for vimentin (**d**) and CD34 (**e**) and negative for S-100 (**f**).

Needle biopsy specimen demonstrated proliferation of bland spindle cells. The cellularity was low (Fig. 2a). There was haphazard proliferation of bland spindle cells with elongated or oval nuclei (Fig. 2b). There was admixture of some adipocytes in the lesion (Fig. 2c). Tumor cells with comma-like shape or wavy nuclei were focally observed (Fig. 2c). Storiform pattern or cartwheel appearance was not obvious. Immunohistochemically, the spindle cells were positive for vimentin (Fig. 2d), CD34 (Fig. 2e). There were few S-100-positive cells (Fig. 2f). MDM2, CDK4, p16, α-SMA, desmin, STAT6, c-kit, β-catenin and AE1/AE3 were negative. Ki-67 index was 1%. Biopsy diagnosis was benign stromal tumor.

**Figure 3:**
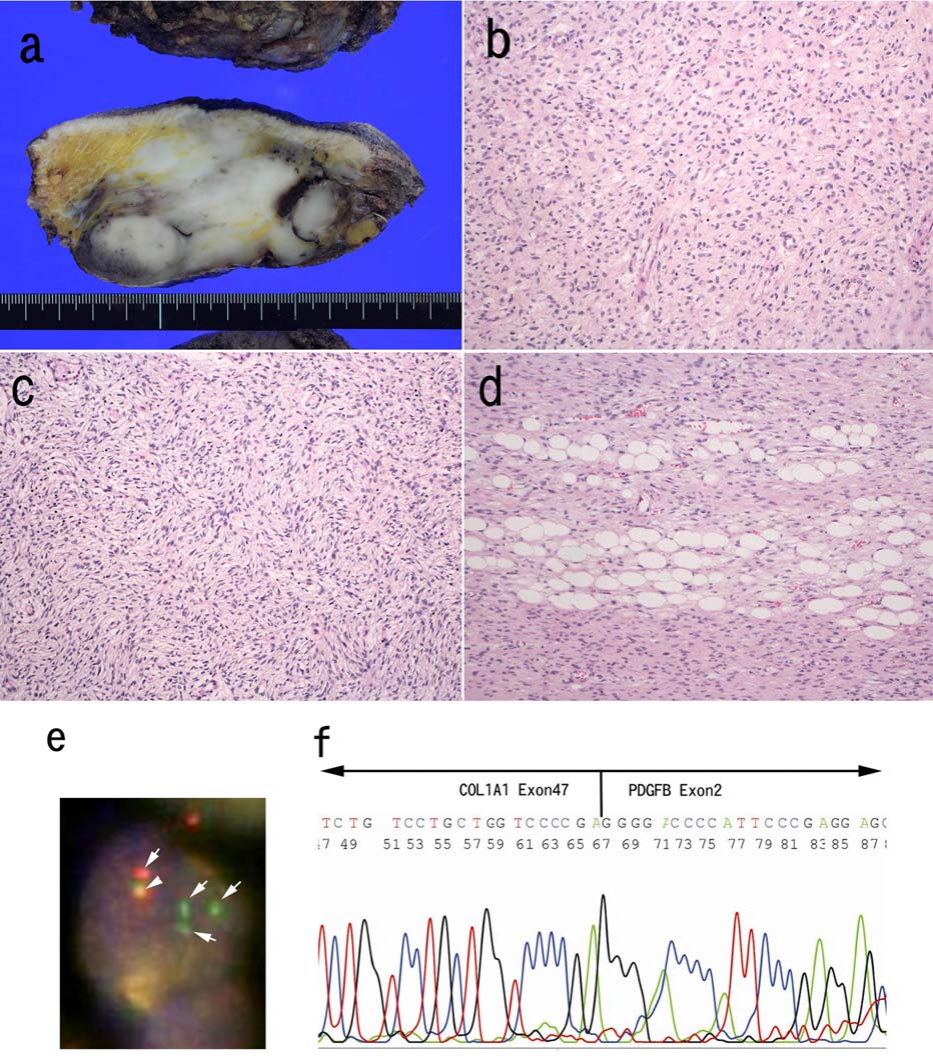
Pathological findings of surgically resected specimen; the cut surface of the tumor was white, solid multinodular mass measuring 83 × 45 mm (**a**); neurofibroma-like morphology was seen similar to that in the biopsy specimen (H&E staining) (**b**); cartwheel pattern was observed at tumor periphery (**c**); tumor cells infiltrated into adipose tissue (H&E staining) (**d**). FISH analysis for PDGFB gene was conducted (**e**). The arrows indicate split signals (green and orange) of the PDGFB gene (e). The arrow head indicates intact PDGFB gene (yellow) (e). Sequence analysis of the RT-PCR product from paraffin embedded specimens confirmed the translocation between COL1A1 exon 47 and PDGFB exon 2 (**f**).

Excision of the tumor was performed 3 months after the biopsy. Macroscopically, white, solid multinodular tumor was situated in the subcutis. There was no involvement of the spinal column with the tumor. The size of the mass was 83 × 45 mm (Fig. 3a). Histologically, neurofibroma-like morphology was predominant (~80%) in the main portion of the tumor (Fig. 3b). Storiform pattern (Fig. 3c) or honeycomb-like fat invasion was only observed at the periphery of the tumor (Fig. 3d). Nuclear atypia was mild. Results of immunohistochemistry were same as the biopsy. We conducted fluorescence *in situ* hybridization (FISH) analysis, which clarified split signals of PDGFB gene (Fig. 3e). Further, reverse-transcription polymerase chain reaction (RT-PCR) using FFPE section showed COL1A1-PDGFB transcript [3]. Subsequent sequence analysis after RT-PCR confirmed the fusion between exon 47 of COL1A1 and exon 2 of the PDGFB gene (Fig. 3f).

The tumor showed local recurrence and neck metastasis 28 months after the resection, and these lesions were resected 33 months after the resection. Furthermore, the tumor showed recurred on the posterior neck again and was excised 63 months after the first resection.

**Table 1:** Previous case reports of stromal tumors harboring COL1A1 exon 47/PDGFB translocation.

Year	Author	Age	Sex	Site	Pathological diagnosis
2021	Our case	31	M	Posterior neck	Neurofibromatous change
2017	Tsuchihashi *et al*. [4]	31	M	Mediastinum	Fibrosarcomatous DFSP
2011	Salgado *et al*. [5]	NA	NA	NA	NA
2011	Salgado *et al*. [5]	NA	NA	NA	NA
2010	Muchemwa *et al*. [6]	29	M	Shoulder	NA
2010	Muchemwa *et al*. [6]	40	F	Buttock	NA
2010	Muchemwa *et al*. [6]	9	F	Buttock	NA
2009	Kabumoto *et al*. [7]	53	M	Occiput	Pigmented DFSP
2009	Llombart *et al*. [8]	41	M	Supraclavicular	Conventional DFSP
2007	Takahira *et al*. [3]	49	M	Groin	Superficial adult fibrosarcoma
2007	Takahira *et al*. [3]	27	M	Groin	DFSP with fibrosarcoma
2007	Takahira *et al*. [3]	30	M	Cheek	Pigmented DFSP
2007	Szollosi *et al*. [9]	NA	NA	NA	DFSP with fibrosarcoma
2003	Gökden *et al*. [10]	25	F	Groin	Storiform/focal myxoid
2003	Gökden *et al*. [10]	37	F	Groin	Storiform/focal myxoid/GCF-like
2003	Sirvent *et al*. [11]	32	M	Infraclavicular fossa	NA
2003	Sirvent *et al*. [11]	2	M	Chest wall	GCF
1998	O’Brien *et al*. [12]	31	F	Upper back	Conventional DFSP
1998	O’Brien *et al*. [12]	43	F	Groin	Conventional DFSP

NA, not available; GCF: giant cell fibroblastoma.

## DISCUSSION

Herein, we report a case of DFSP showing a predominant neurofibroma-like change with difficulty in pathological diagnosis. Detection of COL1A1 exon 47/PDGFB exon 2 chimeric gene provided definite diagnosis.

DFSP is a locally aggressive stromal neoplasm [1]. This tumor usually presents in young to middle-aged adult patients. These neoplasms occur most commonly on the trunk and neck. This tumor often shows local recurrence if the resection is insufficient. In our case, surgical margin was positive and recurred 18 months after resection.

Typical histological findings of DFSP are characterized by diffuse infiltration of the spindle cells into dermis and subcutis with storiform or cartwheel patterns. Invasion to the adipose tissue often show ‘honeycomb-like’ appearance. The tumor cells of DFSP composed of uniform spindled cells containing elongated nuclei. Nuclear pleomorphism is not apparent, and mitotic figure is rare. In our case, the tumor predominantly showed neurofibroma-like morphology, such as bland nuclei or comma-shaped nuclei. Typical storiform pattern or fat invasion was minimum and was observed at the periphery of the deep area. Therefore, this demographic feature of our tumor made the diagnosis difficult in both the biopsy and in the resected specimen.

CD34-positive spindle cell tumor may arise in the posterior side of neck, such as spindle cell lipoma, neurofibroma and DFSP with neurofibromatous change. The former two lesions are benign, while, DFSP is locally aggressive. To avoid underdiagnosis of this tumor, pathologists have to keep neurofibromatous DFSP in mind because wide resection is recommended for the treatment of DFSP. Differential diagnosis of these tumors seems to be easy in almost all cases by the findings of histology based on H&E sections and immunohistochemistry. Nevertheless, we recommend to perform RT-PCR or FISH for a correct diagnosis and an adequate treatment in a challenging case of neurofibroumatous change.

To best of our knowledge, there is only one case series about DFSP with neurofibromatous change, reported by Kovarik *et al*. at 2004 [2]. Kovarik *et al*. reported six cases of DFSP in which initial biopsy specimens showed predominant neurofibromatous changes that caused problems in diagnosis [2]. The location of the lesion was neck in two cases, was abdomen in two cases and the others were on back and scalp. Immunohistochemical examination showed that all cases were positive for CD34. Three cases of their series were S-100 protein-negative. Remaining three cases were comprehensively diagnosed as DFSP by several histologic and clinical characteristics despite S-100 negativity and lack of molecular study.

There are 19 cases of DFSP cases with COL1A1 exon 47/PDGFB chimeric gene in literatures, including our case (Table 1) [3–12]. Histological characteristics were available in 13 cases. Ten cases (77%) of these cases had special histological findings. Although Giacchero *et al*. indicated that there was no correlation with molecular subtype of COL1A1-PDGFB fusion gene and the clinico-histopathological features [13], DFSP with COL1A1 exon 47/PDGFB gene might have high tendency to show unusual morphology.

In conclusion, we described a case of DFSP with neurofibromatous change. Biopsy diagnosis for this lesion may be difficult, especially because of its blunt cellular morphology and uneven demographic distribution of characteristic histological findings of DFSP.
